# Effect of vitamin D on apoptotic marker, reactive oxygen species and human sperm parameters during the process of cryopreservation

**DOI:** 10.22038/ijbms.2019.36258.8634

**Published:** 2019-09

**Authors:** Mahin Taheri Moghadam, Yousef Asadi Fard, Ghasem Saki, Roshan Nikbakht

**Affiliations:** 1 Cellular and Molecular Research Center, Ahvaz Jundishapur University of Medical Sciences, Ahvaz, Iran; 2 Student Research Committee, Ahvaz Jundishapur University of Medical Sciences, Ahvaz, Iran; 3 Fertility, Infertility and Perinatology Center, Imam Khomeini Hospital, Ahvaz Jundishapur University of Medical Sciences, Ahvaz, Iran

**Keywords:** Apoptosis, Human sperm freezing, Infertility, Oxidative stress, Vitamin D

## Abstract

**Objective(s):**

Sperm cryopreservation plays an undeniable role in assisted reproductive technology. However, this process significantly reduces the motility, viability, morphology and nuclear integrity of sperm. Reasons of these changes were oxidative stress and apoptosis. The aim of this study was to evaluate the influence of vitamin D on the survival and integrity of fertile sperm after cryopreservation.

**Materials and Methods:**

Semen sample of 18 males with normal parameters was used. After swimming up, each sample was divided into two parts. 20 µmol vitamin D was added to one part as experimental group and the other part was left untreated as control group. The samples in all groups were frozen for 14 days. Post-thawing, the groups were evaluated for sperm motility, and viability using eosin staining, morphology using the Diff-Quick staining and apoptosis by TUNEL, Annexin-V and caspase-3 activity assay. By using nitrobluetetraxolium test and thiobarbituric acid, the reactive oxygen species (ROS) and lipid peroxidation of sperms were measured, respectively.

**Results:**

In comparison with control groups, motile and viable sperm concentration was substantially higher in treated groups (*P*-value<0.05); however, morphological analysis did not show any remarkable changes. Also, ROS and lipid peroxidation values were dramatically reduced by vitamin D (*P*-value<0.05). TUNEL and Annexin assay for apoptosis were considerably lower in treated groups (*P*-value<0.05), but caspase activity assay revealed no significant difference between groups.

**Conclusion:**

The results have shown that the addition of vitamin D to a freezing medium leads to higher quality and function of human sperm.

## Introduction

Cryopreservation of sperm is used for men who undergo chemotherapy and radiotherapy, treatment cycles with assisted reproductive technology (ART) or sperm ejaculation abnormalities (1). Cryopreservation of sperm is the only method that gives couples the chance to have children after such conditions in the future. It should be noted that this method is known as a cell damage method and has adverse effect on the structure and function of sperm. It can induce ice formation, excessive dehydration, plasma membrane disintegration, acrosome leakage, mitochondrial injury and DNA fragmentation, which have harmful effects on sperm structure and its fertility potential (2-4). After freezing, oxidative stress and reactive oxygen radicals (ROS) can be produced in sperm, and it has been shown that they have some damaging effects on it (5). Several damages such as lipid peroxidation, DNA damage, apoptosis and sperm motility reduction can be induced because of the high susceptibility of spermatozoa to oxidative stress and ROS (6). Nowadays, the main aim of semen cryopreservation technology is to improve an optimal number of functionally intact spermatozoa after thawing (7). In order to control of the level of ROS, and promote motility and survival of sperm, numerous antioxidants have been proven to be beneficial in treating male infertility (8). Antioxidants are molecules that have ability to inhibit or reduce oxidative process in other molecules by scavenging released free radicals (9). There are several antioxidants that are known to improve sperm quality such as vitamin E and C, as well as selenium (Se) and zinc (Zn) (10). As a result, addition of antioxidants to freezing may help spermatozoa against oxidative stress and other damages. Vitamin D is an antioxidant that contributes to numerous functions of the reproductive system (11). In some experiments that have been performed on animals, the role of vitamin D for male reproduction has been demonstrated. Some studies showed that there is a positive association between vitamin D serum level and sperm motility. In addition, they demonstrated that human sperm function may be influenced by vitamin D (12). These observations have led to the hypothesis that vitamin D may have effect on preventing sperm damage induced by cryopreservation in fertile men. So, the aim of this study was to evaluate the effect of vitamin D on apoptosis, ROS and human sperm parameters during the process of cryopreservation.

## Materials and Methods

***Preparing of samples***

Semen sample was collected by masturbation in sterile containers after 3 day of sexual abstinence and transferred to laboratory in university. Semen sample of men with smoking, old age and infertility was excluded from this study.

 In order to extract the normal samples, sperm parameters (volume, concentration, motility and morphology) were evaluated. According to WHO 2010 guidelines, the definition of a healthy man is a person who has the following parameters: volume of semen >2 ml, sperm concentration >15 × 10^6^ ml^-1^, normal morphology >4%, and sperm motility>40%. After routine semen analysis and in order to remove supernatant, the samples were washed twice by culture medium (Hams F10) (Biochrom.K.G) and centrifuged at 300 × g for 5 min. Then, 1 ml culture medium was added and swimming up was performed in incubator at 37 ^°^C for 45 min. Then, the supernatant was divided into two groups: experimental group with 20 µmol vitamin D and control group without vitamin D. Sperm freezing medium (CE 0086) was added to the groups in a proportion of 1:1 in a dropwise manner. After 15 min of equilibration at room temperature, the mixture was frozen in liquid nitrogen vapor for 15 min. Then, they plunged into liquid nitrogen (-196) in cryotube for storage (13). Samples were cryopreserved for at least two weeks. For thawing, samples were removed from liquid nitrogen, thawed at room temperature for 20 min and incubated at 37 ^°^C for 20 min. The cryoprotective medium was removed by centrifugation (1800 g in 5 min) and the pellet was suspended in a suitable volume of culture medium. Afterwards, samples were analyzed for motility, viability, apoptosis marker and oxidation assessment. 

**Figure 1:**
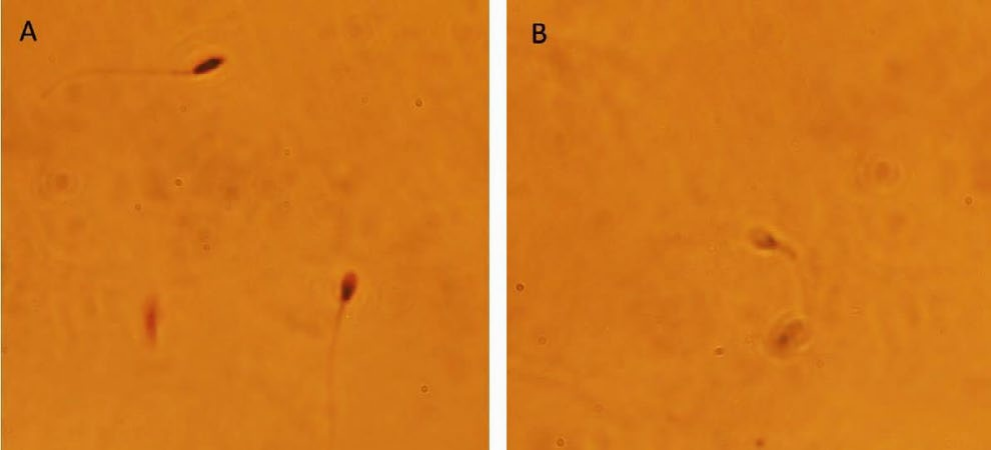
Effect of 20 µmol vitamin D on human sperm motility after freezing-thawing process. a (rapid progressive), b (slow progressive), c (non-progressive) and d (immotile).*: *P*-value <0.05

**Figure 2:**
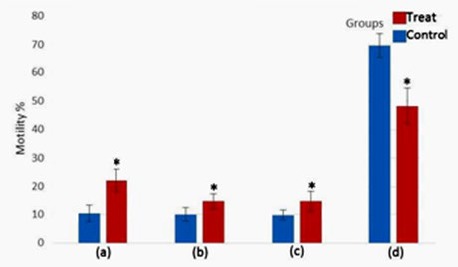
Evaluation of human sperm viability using eosin staining; (A) stained (red) dead spermatozoa, (B) unstained (white) intact spermatozoa

**Figure 3:**
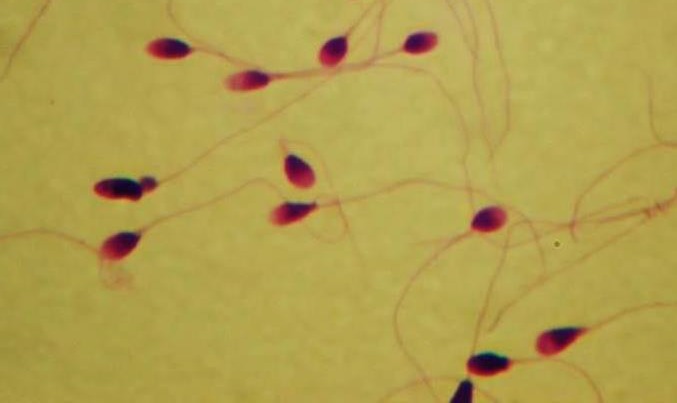
Effect of 20 µmol vitamin D on human sperm viability after freezing-thawing process. *: *P*-value<0.05

**Figure 4:**
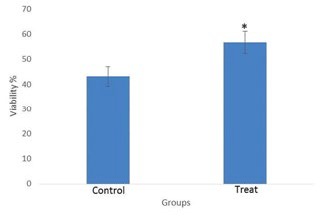
Morphology assessment of sperm using Diff-Quick staining. A spermatozoa with a smooth oval configuration with a well-defined acrosome, no defects of neck, midpiece of tail and no cytoplasmic droplets >50% the size of head is normal

**Figure 5:**
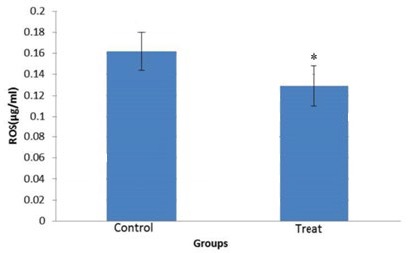
Effect of 20 µmol vitamin D on reactive oxygen species of human sperm after freezing-thawing process. * *P*-value <0.05

**Figure 6:**
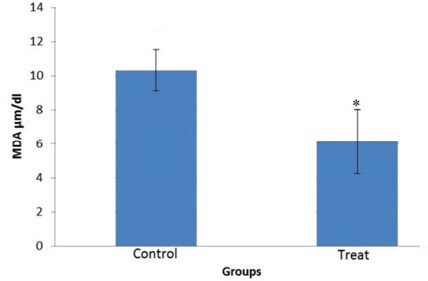
Effect of 20 µmol vitamin D on malondialdehyde concentration of human sperm during cryopreservation. * *P*-value<0.05

**Figure 7:**
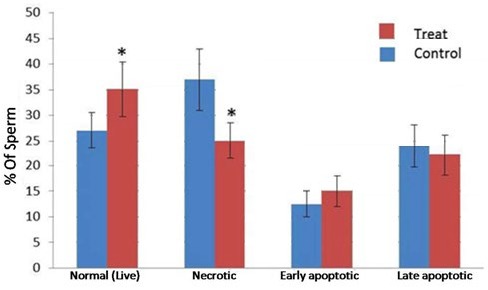
Effect of 20 µmol vitamin D on apoptosis in human sperm after freezing-thawing process. *:* P*-value <0.05

**Figure 8:**
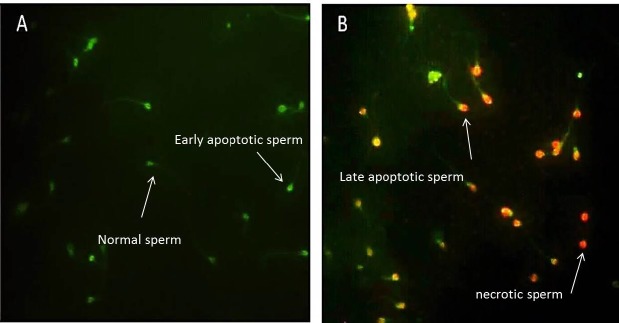
Detection of apoptosis in spermatozoa using Annexin˗V staining. (A). Spermatozoa with no sign of phosphatidylserine translocation [Annexin˗V (˗)/ propidium iodide (PI) (˗)] (pale green) as a normal cell and cells with green color were as early apoptotic cells [Annexin˗V (+)/PI (˗)], (B) late apoptotic cell.[Annexin˗V(+)/PI(+)]. Annexin˗V staining (green) and PI staining (red).necrotic cell [Annexin˗V (˗)/PI (+)](only red)

**Figure 9:**
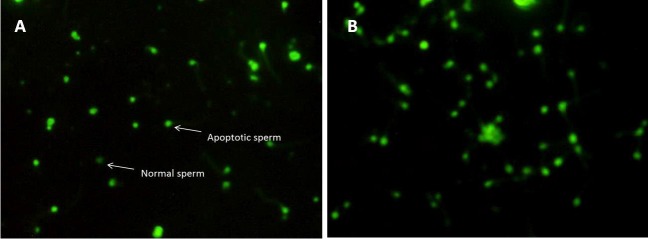
Evaluating the sperm DNA fragmentation using TUNEL test. Dark green cells are abnormal spermatozoa and pale green are normal spermatozoa. (A) treated group.(B) control group

**Figure 10:**
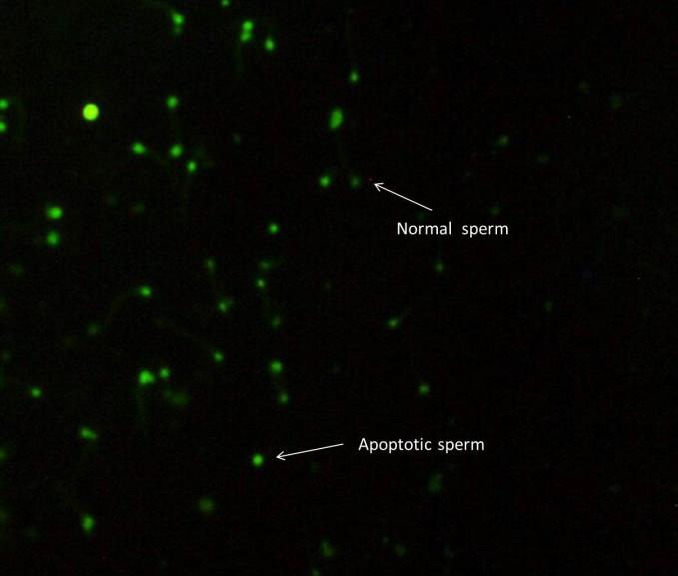
Effect of 20 µmol vitamin D on apoptosis (DNA fragmentation) in human sperm after freezing-thawing process. *: *P*-value <0.05

**Figure 11:**
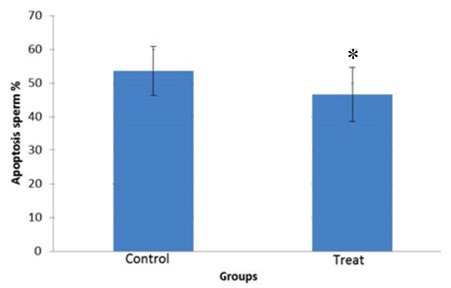
Immunostaining for active caspase-3 in human sperm. Sperm with intense green fluorescence is positive for caspase-3 activity (apoptotic sperm) and pale green for normal sperm

**Table 1:** Characteristics of 18 fresh human semen

Normal morphology (%)	Total motility (%)	Concentration (million/ml)	Volume (ml)	Parameters of sperm
51.11±13.86	53.22±1.04	93.66±65.67	2.65±0.53	Mean ± SD

***Sperm motility***

After thawing, the motility of sperm was evaluated by light microscope at 400 magnifications. The motility of sperm was categorized into rapid progressive (type a), slow progressive (type b), non-progressive (type c) and immotile sperm (type d) according to the WHO laboratory manual for semen parameters (WHO 2010 guidelines). According to this category, 100-200 sperms were counted and total sperms with type ‘a+b+c’ motility were reported as the total motile sperms (Table 1) (14).

***Sperm morphology***

Sperm morphology assessment was performed by the Diff-Quick staining method. After smear preparation, smear was stained with the Diff-Quick staining kit. By using a microscopy at magnification ×400, morphology of sperm was checked. According to the WHO standards, a normal sperm is a sperm that has a smooth oval configuration head with a well-defined acrosome (involving 40-70% of the sperm head) and it has no defects of neck, midpiece or tail and no cytoplasmic droplets >50% the size of the sperm head. By these criteria, if 4% sperms include these features, the samples are known as a normal morphology (14,15).

***Sperm viability***

In order to determine the percentage of viable sperm, the eosin-nigrosin staining was performed. For this purpose, 20 µl of eosin was added to 20 µl of thawed sample. Then, a smear was prepared on glass slide and was observed under a microscope at ×400 magniﬁcation. This staining is based on permeability of the dye into the damaged plasma membrane. In this method, intact sperm is unstained and dead sperm is red. By counting 200 sperm on each slide, the viability of sperm was examined and the percentage of intact sperm was defined (16, 17).

***Annexin-*** ***V-FITC/PI assay***

Phospholipids are distributed asymmetrically between inner and outer leaflets of the plasma membrane of live cells. In early apoptosis, this asymmetry is disrupted and on the outside surface of the plasma membrane, the phosphatidylserine (PS) becomes exposed. The translocation of PS to the outer layer of plasma membrane was detected by the Annexin-V fluos staining kit (NO 11 858 777 001, Roche, Mannheim, Germany). As a manufacture protocol, 300 µl from each sample was washed with phosphate-buffered saline (PBS) and diluted in 100 µl Annexin-V buffer, 2 µl Annexin-V-FITC and 2 µl propidium iodide (PI) solutions. At the room temperature and in the dark, the samples were incubated for 20 min. Then, by using a fluorescence microscopy at ×400 magnifications, smear was observed. In this method, at least 100 sperm was counted and divided into four groups as 1) intact cell (pale green or Annexin˗V (-)/PI (˗), 2) early apoptotic cell (dark green or [Annexin˗V (+)/PI (˗)]) , 3) late apoptotic cell (both red and green or [Annexin˗V(+)/PI(+)].), and 4) necrotic cell (red or [Annexin˗V (˗)/PI (+)])(18).

***TUNEL assay***

Evaluation of sperm DNA fragmentation was performed using the terminal deoxynucleotidyltransferase (TdT) - mediated deoxyuridinetriphosphate (dUTP) nick-end labeling (TUNEL) assay (*In Situ* Cell Death Detection Kit, POD, NO 11 684 817 910, Roche, Penzberg, Germany). Briefly, methanol-fixed smears were plunged to PBS for 30 min. Then, smears were incubated with H_2_O_2_ %3 in methanol for 15 min and plunged in PBS. For membrane permeability, smears were incubated with Triton 0.03% in 4 ^°^C for 15 min and washed with PBS. Then, the reaction was performed by incubating the sample in 45 µl labeling solution containing 5 µl TdT enzyme solution and dUTP for 1.5 hr at 37 ^°^C. Afterward, the samples were washed with PBS for stopping the enzyme reaction. The smears were observed with invert fluorescent microscope at ×400 magnifications. At least 100 sperm were counted and divided to intact sperm (pale green or low level of fluorescence) and apoptotic sperm (dark green or intense fluorescence). Samples without 5 µl TdT were assessed to negative control (19).

***Immunocytochemistry for caspase 3 activation***

In order to detect apoptosis, we used a purified rabbit anti-active caspase-3 monoclonal antibody, which specifically recognizes the active form of caspase-3 in human. In this assessment, paraformaldehyde-fixed smears were washed twice with PBS, and incubated for 1 hr in blocking buffer (10% NGS, 0.4% Triton X-100 in PBS) at room temperature. Then, they were incubated for 1 hr with primary antibody (diluted 1/1000) and buffer (2% NGS, 0.4% Triton X-100 in PBS). The cells were washed 3 times with 0.1% Tween-20 in PBS, and the bound antibodies were detected with biotinylated anti-rabbit immunoglobulin antibodies (Amersham; diluted 1:200 in incubation buffer). The cells were then examined by fluorescent microscope at×400 magnification. A minimum 100 sperm were counted into two groups as intact sperm (low level fluorescence or pale green) and apoptotic sperm (intense fluorescence or dark green). Samples without any primary antibody were assessed to confirm the absence of nonspecific staining (negative control)(20).

***Measurement of reactive oxygen species***

ROS was studied using the nitro blue tetrazolium (NBT) test. For NBT solution, 10 mg of nitrobluetetrazolium chloride powder (N6876-Sigma) was added to 10 ml PBS (0.1%) and stirred at room temperature for 1 hr. In this method, 200 µl of sample was washed twice by PBS and centrifuging at 300 × g for 5 min. The sample was resuspended in 100 µl PBS and an equal volume of 0.1% NBT and then shaken at 37 ^°^C for 45 min. In this incubation, the intracellular ROS of sperms produce crystals that were solubilized in 60 µl each of 2 mol l-1 KOH and dimethyl sulfoxide (DMSO). After 5 min, the color of mixture was measured by an ELISA reader (BIO RAD- model680) at 655 nm (21).

***Measurement of lipid peroxidation***

Malondialdehyde (MDA) levels were analyzed according to the thiobarbituric acid (TBA) methods. After thawing, 0.5 ml of sample was subjected to rapid freeze-thawing three times to lyse the cells. The spermatozoa were separated from the sample by centrifugation (1000 g for 10 min at room temperature). The supernatant was used for the determination of seminal MDA. To each tube, 0.5 ml of TBA (0.67 g of 2- TBA dissolved in 100 ml of distilled water with 0.5 g NaOH and 100 ml glacial acetic acid added) was added and then heated for 1 hr in a boiling water bath. After cooling, each tube was centrifuged for 10 min at 4,000 × g and the supernatant absorbance was read on a spectrophotometer at 532 nm (21).

## Results

Total samples were obtained from 18 fertile men. Samples characteristics before cryopreservation were shown in Table 1.

***Sperm motility assessment***

The effect of vitamin D on the sperm motility of frozen-thawed human sperm is presented in Figure 1. Treated groups with 20 µmol vitamin D had higher motility in a, b and c grade compared to control groups (*P*<0.05). Rapid progressive spermatozoa (group “a”) had a mean value of 22.21±3.87 in treated group and a mean value of 10.37±2.88 in control group (*P<*0.05). The slow progressive spermatozoa (group “b”) had a mean 14.78±2.77 in treated group and 10.18±2.37 in control group (*P<*0.05). The non-progressive motile spermatozoa (group “c”) had a mean 14.73±2.77 in treated group and 9.85±1.79 in control group (*P<*0.05). These observations suggest that addition of vitamin D to freezing medium could improve the motility of the frozen-thawed human spermatozoa.

***Sperm viability***

The observations of eosin staining were classified into 2 groups: Dead sperm (red color) and intact sperm (unstained) (Figure 2). Sperm viability had a mean 56.77±4.4 in treated group and 43.22±4.08 in control group. Sperm viability in the vitamin D-treated group was significantly higher than control group (*P-*value<0. 05) (Figure 3).

***Sperm morphology***

Percentage of sperms with normal morphology in the vitamin D-treated groups was more than control group (53.97±12.77 vs 46.03±10.77), but it was not significant (Figure 4).

***Reactive oxygen species***

ROS value in vitamin D-treated group was significantly lower than control group (*P-*value <0.05). This level in treated groups was 0.129±0.019 and in control group was 0.162±0.018 (Figure 5).

***Lipid peroxidation***

The mean concentration of MDA in vitamin D-treated group was significantly lower than control group (*P-*value <0.05). This level in treated group was 6.158±2.008 and in control group was 10.336±1.2 (Figure 6).

***Annexin-V assay***

Annexin-V assay was performed for evaluation of membrane integrity and apoptosis in sperm. The results were classified into four groups: normal, early apoptosis, late apoptosis, and necrosis. The results of this method were as follows: the percentage of normal sperm in vitamin D-treated group was higher than control group (*P-*value<0. 05) and the percentage of necrotic sperm cells was lower in vitamin D-treated group (*P-*value <0.05). Percentage of early and late apoptosis sperm cells was lower in vitamin D-treated group but was not significant compared to control group (Figure 7, 8).

***DNA fragmentation***

Spermatozoa DNA damage was significantly lower in vitamin D-treated group (*P-*value<0.05). The mean values of apoptotic spermatozoa were 46.77±11.79 and 53.72±7.25 in treated and control groups, respectively (Figure 9, 10). These results suggest that addition of vitamin D to freezing medium can reduced DNA fragmentation.

***Immunocytochemistry of caspase-3 activity***

Post-thaw caspase-3 activity assay revealed no significant differences between vitamin D-treated and control groups. Mean value of sperm with active caspase-3 was 47.88±7.4 in vitamin D-treated group and 52.22±7.4 in control group (Figure 11).

## Discussion

The objective of the present study was to determine whether addition of vitamin D to freezing medium could improve the quality of frozen-thawed spermatozoa in humans, and how vitamin D protects spermatozoa against damages during the process of cryopreservation. In this study, it was found that supplementation of freezing medium with vitamin D at a concentration of 20 µmol significantly improved post-thaw motility, integrity of cell membrane and DNA, apoptosis, ROS, post-thaw motility, and viability of human sperm. The process of cryopreservation exposes spermatozoa to many physical and chemical damages. It causes to generate ROS and reduce antioxidant defenses in spermatozoa (22-24). High ROS concentrations can disturb plasma membranes integrity, DNA stability and other parameters of sperm (25). In this study, the results showed that during freezing and thawing methods, the rate of ROS was significantly increased in control group and supplementation of vitamin D with cryopreservation causes to reduce ROS generation. Similar results have been reported by some researchers. Findings of similar researches have shown that vitamin C supplementation reduced ROS generation (26,27). There are several evidences supporting the antioxidant activity of vitamin D (cholecalciferol) in the oxidative stress. The results in some experimental studies implied that vitamin D administration in diabetic mice helps to reduce the ROS formation by suppression of the gene expression of NADPH oxidase (28). NADPH oxidase is a main resource of ROS, and its activation contributes as a positive marker for oxidative stress (29). Also, the results of another study showed that vitamin D could improve the superoxide dismutase (SOD), and enzymatic antioxidants activity in mice (30). So, vitamin D as an antioxidant may be beneficial for cryopreservation of spermatozoa.

On the other hand, some stresses during cryopreservation could damage the plasma membrane integrity (2) and cause an early event of apoptosis in human spermatozoa (31). Plasma membrane integrity is the main parameter in the evaluation of sperm functionality (32). Phospholipids that are distributed asymmetrically between the inner and outer leaflets of the plasma membrane of live cells are very important for membrane integrity. During early apoptosis, this asymmetry is disrupted and PS becomes exposed on the outside surface of the plasma membrane (31). In the present study, the plasma membrane integrity damage and early apoptosis of sperm was significantly higher after cryopreservation procedure in control group, but supplementation of vitamin D with the freezing medium resulted in significant increase of the percentage of post-thaw spermatozoa with intact membrane. These data are in agreement with the findings of Hu *et al*. (33). They demonstrated that adding antioxidant such as vitamin E to freezing-medium improved the percentages of intact membrane. Some studies showed that vitamin D can act in the defense system of cell phospholipid membrane and mitochondrial sheath against oxidative stress and increase the production of ROS-collecting enzymatic antioxidants (34). In another study, Wiseman and colleges reported that the hydrophobic parts of vitamin D could interfere with fatty acid residues that impair the viscosity of cell membrane and thus protected the cell membrane from lipid peroxidation and the harmful effects of free radicals (35).

Oxidative stress was studied as a possible mechanism for the origin of sperm DNA damage (36). Failure to detoxify these agents can result in oxidative damage and, at the nuclear level, lead to production of basic sites, purine or pyrimidine oxidation and DNA strand breakage (37). Our investigation showed that 20 µmol vitamin D has a beneficial effect on the reduction of DNA damage induced by cryopreservation. Eghbali and colleges performed an investigation on buffalo bulls and they found that 1 and 2 mg/ml of selenium can increase semen antioxidants activity and reduce DNA damage after freezing and thawing procedure (38). 

On the effect of cryopreservation on apoptosis, some studies have revealed that apoptosis markers increase in spermatozoa following freezing and thawing. These studies also showed that apoptosis was increased by DNA fragmentation and PS externalization in freezing sperms and vitamin D could improve these factors in sperms. Active caspase-3 was detectable in this study. Vitamin D may control caspase activation in apoptosis (39), but in our study vitamin D did not have any significant effect on caspase activation. Wundrich and colleges have shown that active forms of caspases 1, 8 and 9 were detected significantly after cryopreservation of sperms, but caspase-3 was not expressed very significantly. Therefore, due to the poor expression of caspase-3 during cryopreservation, it has not been affected by vitamin D (40). About sperm parameters during freezing and thawing, cryopreservation has negative effect on sperm motility, viability and morphology. Sperm motility is especially sensitive to damage during cryopreservation (41). Our data were similar to other studies. The results of similar studies showed that cryopreservation caused to decrease sperm motility and viability (42, 43). In this study, post-thaw human sperm motility was significantly improved by the addition of vitamin D, which is in agreement with the previous reports. Taylor and colleges showed that the different doses of vitamin E were associated with improved post-thawing human sperm motility (44). Florin *et al*. freezed boar semen with the addition of vitamin E and C supplements and they observed that these supplements increased sperm motility after thawing (45). Post-thaw sperm viability was significantly improved in treated groups by addition of 20 µmol vitamin D, which is in agreement with study of Breininger *et al.* and Pena *et al*. They found that supplementation of vitamin E with the cryopreservation media prior to cryopreservation significantly enhanced survival rate of post-thaw boar spermatozoa (46, 47). And also, our result showed that sperm morphology improved by vitamin D in treated group compared to control group but it was not significant.

## Conclusion

Addition of vitamin D on freezing media protects human sperms from oxidative stress during cryopreservation method and improves motility, viability, integrity of membrane and DNA sperm cells.
